# Prognosis of chronic Chagas heart disease and other pending clinical challenges

**DOI:** 10.1590/0074-02760210172

**Published:** 2022-06-06

**Authors:** Rosália Morais Torres, Dalmo Correia, Maria do Carmo Pereira Nunes, Walderez O Dutra, André Talvani, Andréa Silvestre Sousa, Fernanda de Souza Nogueira Sardinha Mendes, Maurício Ibrahim Scanavacca, Cristiano Pisani, Maria da Consolação Vieira Moreira, Dilma do Socorro Moraes de Souza, Wilson de, Silvia Marinho Martins, João Carlos Pinto Dias

**Affiliations:** 1 Universidade Federal de Minas Gerais, Belo Horizonte, MG, Brasil; 2 Universidade Federal do Triângulo Mineiro, Uberaba, MG, Brasil; 3 Universidade Federal de Ouro Preto, Ouro Preto, MG, Brasil; 4 Universidade Federal do Rio de Janeiro, Rio de Janeiro, RJ, Brasil; 5 Fundação Oswaldo Cruz-Fiocruz, Instituto Nacional de Infectologia Evandro Chagas, Rio de Janeiro, RJ, Brasil; 6 Universidade de São Paulo, Instituto do Coração, São Paulo, SP, Brasil; 7 Universidade Federal do Pará, Belém, PA, Brasil; 8 Universidade de Pernambuco, Recife, PE, Brasil; 9 Fundação Oswaldo Cruz-Fiocruz, Belo Horizonte, MG, Brasil

**Keywords:** Chagas disease, Chagas cardiomyopathy, prognosis, evolution markers, public health

## Abstract

In this chapter, the main prognostic markers of Chagas heart disease are addressed, with an emphasis on the most recent findings and questions, establishing the basis for a broad discussion of recommendations and new approaches to managing Chagas cardiopathy. The main biological and genetic markers and the contribution of the electrocardiogram, echocardiogram and cardiac magnetic resonance are presented. We also discuss the most recent therapeutic proposals for heart failure, thromboembolism and arrhythmias, as well as current experience in heart transplantation in patients suffering from severe Chagas cardiomyopathy. The clinical and epidemiological challenges introduced by acute Chagas disease due to oral contamination are discussed. In addition, we highlight the importance of ageing and comorbidities in influencing the outcome of chronic Chagas heart disease. Finally, we discuss the importance of public policies, the vital role of funding agencies, universities, the scientific community and health professionals, and the application of new technologies in finding solutions for better management of Chagas heart disease.

**Chagas disease and Chagas heart disease: a brief historical review**

When Carlos Chagas identified the *Trypanosoma cruzi* in 1908 and the first human case in April 1909, he turned his attention to cardiac disorders detected in acute cases. As early as 1911, in a conference at the National Academy of Medicine, he detailed this heart disease (studied by him together with pathologists Vianna and Crowell), with attention to the occurrence and severity of myocarditis. The very serious conditions of heart failure, sudden death and arrhythmias that he found in young adults in the region of Lassance, Minas Gerais^1^ attracted his attention and was attributed to schizotripanosis because he was aware of homes infested by the triatomine. Carlos Chagas did not find trypanosomes in the fresh blood test of these patients but in the xenodiagnosis and in subinoculations of the laboratory. For Chagas, heart disease marked the existence of a chronic phase of the disease, which he understood to be the most important event of this “new morbid entity of man”, and expanding his research, he published a broad review in 1916.^2^ He joined Eurico Villela in clinical data studies on heart disease^3^ and, together using clinical reasoning and graphic records with polygraphs attached to the shock of the tip and the jugular pulse, demonstrated the presence of atrioventricular dissociation, complex ventricular arrhythmias, atrial fibrillation and major cardiomegalies, indicating a reserved prognosis. In 1929, Evandro Chagas joined the team, developing pioneering studies with electrocardiograms. Intraventricular blocks and areas of electrical hypoactivity were then described, which later turned out to be signs of cardiac fibrosis or parietal aneurysms and a poor prognosis.^4^ In short, Carlos Chagas anticipated the scientific findings and pathophysiological conceptions of cardiopathy decades earlier, aspects that remain a challenge to cardiology.

Prognostic studies continued in Brazil, Venezuela and Argentina. Between 1940 and 1960, serological-electrocardiographic surveys were improved, which demonstrated the disease burden and its natural history, a very important element for making political-administrative decisions in the extensive fight against triatomines and transfusion blood control. Old mercurial diuretics and classic digitaline were gradually replaced, and echocardiography proved to be an element of great diagnostic and prognostic value. Then, the Holter system, stress tests, biomarkers of congestive heart failure (CHF) and other diagnostic tools complemented the arsenal of assessing myocardial status, the degree of CHF, the profile of arrhythmias and possible thromboembolic conditions.^5^

Clinical findings in Chagas heart disease, especially in its chronic stage, will shape its diagnosis, prognosis and clinical-social management. Discrete electrocardiographic findings, absence of cardiomegaly and signs of CHF are generally associated with a good prognosis “quod vitam” but are entitled to continued medical attention, given the possibility of evolution.^5^ ^,^ ^6^

The management of Chagas cardiomyopathy was improved after 1960, with the advent of pacemakers, the emergence of new serological techniques and more effective drugs for the stabilisation of arrhythmias and CHF that prolonged the life of Chagas patients and their social insertion. Cardiopathy is better understood as a product of several elements rooted in *T. cruzi* infection modulated by different factors (genetic, environmental, vagus-sympathetic balance, age, gender, race, coinfections, social status, etc.). It was found that the severity of chronic heart disease is directly related to the occurrence and intensity of heart disease in the acute phase,^6^ and in the anatomical-physiological field, myocytolysis, fibrosis and neurovegetative dysautonomy have been considered the main basis of severe disease, factors that still challenge doctors and researchers today.^7^ ^,^ ^8^

**Pathogenesis of chronic Chagas heart disease: new horizons**

It is widely known that Chagas cardiomyopathy is the most severe manifestation of the disease, which develops in up to 30% of infected individuals over the years to decades after the initial infection.^9^ ^,^ ^10^ The clinical presentation varies widely according to the severity of myocardial dysfunction, ranging from asymptomatic to severe forms, with heart failure, cardiac arrhythmias, thromboembolism events or sudden death.^10^ ^,^ ^11^ ^,^ ^12^ The factors associated with different degrees of cardiomyopathy severity are still under investigation.

In fact, even today, the pathogenesis of chronic Chagas cardiomyopathy is not fully understood.^13^ Growing evidence indicates that parasite persistence is central to the disease by driving the immune response, tissue destruction and chronic inflammation. However, *T. cruzi* antigens remain scarce and are not always associated with inflammatory foci in the myocardium^14^. The persistence of the parasite in tissues associated with poorly adapted homeostatic mechanisms, such as the oxidative/antioxidative and proinflammatory/anti-inflammatory processes associated with the dysregulation of the immune response, seems to be essential for the onset and progression of Chagas heart disease.^11^ ^,^ ^12^ Recently, studies using hypercaloric diets in animal models have shown an increase in the parasitic load in adipose tissue with a consequent reduction in parasitaemia in cardiac tissue, suggesting that adipose tissue could act as a reservoir for later migration of the parasite to other organs and a possible protective role of adipose tissue in the evolution of chronic Chagas cardiomyopathy.^13^ ^,^ ^14^

The discrepancy between the severity of tissue damage and parasite load has led to the suggestion that the inflammatory immune response of the host is the most important determinant of progression.^13^ The balance between immune-mediated parasite containment and damaging inflammation of host tissues likely determines the course of disease.^12^ Other pathogenic factors involved include damage to cardiac parasympathetic neurons and coronary microvasculature disorders.^15^ Pathogenic differences in *T. cruzi* strains and host susceptibility are also likely to play a role in clinical patterns and disease severity.^16^ Given the complex interplay between parasites and the host immune response, it is likely that a combination of these and other factors, including host genetic susceptibility and environmental factors, contribute to cardiac disease.^17^ Therefore, the clinical expression of Chagas disease and its prognosis seem to result from multiple factors linked to the parasite, the host and the interaction between them.

The histopathological features of Chagas cardiomyopathy are characterised by a chronic inflammatory process in all cardiac chambers, with focal thickening, tissue destruction and interstitial fibrosis, either focal or diffusely distributed.^18^ Overall ventricular functional impairment is a consequence of the progressive destruction of cardiac fibres and intense fibrosis. Additionally, widespread destruction of myocardial cells, diffuse fibrosis and scarring of the conduction system predispose patients to the frequent occurrence of atrioventricular blocks, intraventricular blocks and sinus node dysfunction in Chagas heart disease. This chronic activation of inflammatory factors in the myocardium seems to determine a worse prognosis for this disease than heart diseases of other aetiologies.^19^ ^,^ ^20^

**Evolution markers of Chagas heart disease**

*Immunological and genetic biomarkers* - After over 110 years of Chagas disease discovery, the identification of reliable markers of treatment efficacy, as well as of the prognosis of chronic Chagas cardiomyopathy (CCC) development, remain major challenges, driving the search for “immunological biomarkers” to anticipate the progression to CCC and thus allowing for better clinical management and/or pharmacological intervention for the benefit of Chagas patients^21^ ^,^ ^22^ In addition, with advances in genomics, “genetic biomarkers” have also been investigated. While immunological and genetic markers present unquestionable potential, the complexity behind each target molecule requires careful and precise integration with well-established clinical parameters (e.g., X-ray, ECG, echo, MRI and clinical assessments) for their validation as clinically actionable markers. Thus, many target molecules have been studied, but few biomarkers of CCC have been proposed to date.

Immune cells recognise, interact, and initiate a cascade of events meant to control the parasite and to regain homeostasis. However, the persistence of this host-parasite interaction initiates a new set of biological events leading to outcomes in the immune/neuro/cardiovascular context. These complex interactions lead to an imbalance in the production of several molecules involved in the inflammatory response and in disease pathology. Thus, the excessive or decreased production of mediators, such as cytokines, chemokines, adhesion molecules, and cellular activation markers, among others, culminates in a potential worsening or stabilisation of CCC and thus has been studied as a biomarker of CCC establishment or progression.

Despite several candidates, an important issue is that validation of prognostic biomarkers must consider several variables that can influence their expression, age, ethnicity, gender, geographical origin of the infected individuals and a clearly defined endpoint. The use of unproperly defined and validated biomarkers could lead to false results that, if employed for clinical management, could lead to harmful instead of beneficial consequences. For instance, TNF is a classical cytokine released by phagocytic cells upon *T. cruzi* invasion, as well as by activated lymphocytes, which is responsible for orchestrating chemokine production and cell activation, intensifying systemic and local inflammatory responses.^23^ ^,^ ^24^ ^,^ ^25^ ^,^ ^26^ During the chronic phase of the disease, TNF has been shown to be an essential key marker for the prognosis of cardiac disturbances in CCC individuals,^27^ ^,^ ^28^ ^,^ ^29^ but not every study supports this positive correlation between TNF levels and electrofunctional deficits in the CCC.^30^ It is not a matter of who is “right or wrong” but which intrinsic variables can interpose the final clinical understanding. Thus, a clear identification of CCC prognostic biomarkers is still a challenging puzzle. To achieve this goal, it is imperative that studies test potential candidates and consider a: the geographical and ethnic origin of the Chagas populations, b: variables such as age and sex, and c: well-established and homogeneous clinical endpoint. In addition, longitudinal studies assessing the expression of different candidate molecules and genes are critical to identify reliable biomarkers of disease progression. A global scientific effort and proper funding are important hindrances to overcome to achieve such goals (Table I).

**TABLE I:** Potential biomarkers for Chagas cardiomyopathy prognosis

Biomarkers	Relevance and/or validity in chagasic cardiomyopathy (CC)	References
Tumor necrosis factor (TNF), atrial natriuretic peptide (ANP), brain natriuretic peptide (BNP) and pro-BNP	↑ BNP, TNF, and pro-BNP or TNF-related to depression of LVEF, with ↑ of LV end-diastolic diameter and with LV premature complexes ↑ ANP and ↑ BNP correlated with worsening ECHO parameters, predict death and demand for heart transplant ROC-based analyses: BNP (280.4 pg/ml) with 96% of sensitivity and 75% of specificity for predicting E/E’ >15 BNP (60 pg/ml or more) has sensitivity of 91.7% and specificity of 82.8% for LV dysfunction prediction.	(31,32,33,34,35)
ɣ-interferon (IFN-**γ**), IL-1, IL-6, IL-12, IL-17andIL-13. Creatine-kinase (CK-MB) isoenzyme Matrix metalloproteinase (MMP-2 and MMP-9); tissue inhibitor of the MMP (TIMP-2)	↑ levels IFN-γ IL-6, IL-12, IL-13, CK-MB, MMP-2, MMP-9, TIMP-2 and (↓ IL10 and IL-17) related to severe CC ↑MMP-9 as for late fibrosis and severe cardiac remodeling ROC-based analyses: ↑MMP-2/MMP-9 ratio related to ECG abnormalities.	(36,37,38,39,40) (41,42,43,44,45)
Vinculin, plasminogen and NK/CD8^+^ T-cell MiR-19a-3p, miR-21-5p, and miR-29b-3p CC - CXC chemokine receptors (CXCR3, CCR4, 5, 7 and 8) and ligandsCXCL9 and 10) CD1D and Programmed cell death (PD-1/PDL1)	↑ myosin (light chain 2 and heavy chain 11), ↑ levels of vinculin and plasminogen and ↑ NK/CD8^+^T-cell correlated to the cardiac dysfunction ↑ expression of MiR-19a-3p, miR-21-5p, and miR-29b-3p correlated with cardiac dysfunction and fibrosis ↑ CXCL9, CXCR3, CCR4, CCR5, CCR7, CCR8, CD1D and PD-1/PDL1 expression correlated with myocarditis and/or LV worsening ↓ genotypic frequencies of CXCL9 (rs10336*CC)* and*CXCL10*(rs3921*GG)*, and ↑*CCR5*(rs1799988*CC) correlated with LV dysfunction.*	(46,47,48,49)(50,51)

LV: left ventricle; EF: ejection fraction; (E/E) early diastolic mitral annular tissue velocity ratio; ECG: electrocardiogram; ECHO: echocardiogram.

*Electrocardiographic markers* - Electrocardiography has been considered an important tool in the management of patients with Chagas disease, and the presence of typical electrocardiographic abnormalities, which encompass a wide spectrum of presentations, is needed for the recognition of the cardiac chronic form of the disease. This condition must indicate the need for more cardiac evaluation and closer follow-up.^52^

The initial manifestations of Chagas cardiomyopathy are generally mild, and most patients have asymptomatic ECG abnormalities. The earliest signs are typically conduction-system defects, especially right bundle branch block alone or with left anterior fascicular block.^53^ ^,^ ^54^ ^,^ ^55^ ^,^ ^56^ Premature ventricular contractions are a common finding that may be missed on 12-lead ECG, usually detected by Holter monitoring or stress testing. Indeed, Chagas cardiomyopathy is a highly arrhythmogenic condition that is characterised by sinus and junctional bradycardias, atrial fibrillation or flutter, atrioventricular blocks, and nonsustained or sustained ventricular tachycardia.^57^

The predominant distribution of fibrosis to the posterior and apical regions of the left ventricle, involvement of the sinus node and electrical conduction system distinguishes Chagas cardiomyopathy from other cardiomyopathies.^52^ Electrocardiographic changes in the late disease stage are also pronounced, with conduction disturbances and arrhythmias occurring more frequently than in dilated cardiomyopathies of other aetiologies.^58^

In a cohort of more than 260 thousand patients, of whom 7590 had Chagas disease, 38.8% of those with other heart diseases had an altered electrocardiogram, whereas 68.5% of patients with chronic Chagas heart disease exhibited electrocardiographic changes. The prevalence of these changes increased with age, and between 60-69 years of age, 74% of CCC patients had an abnormal ECG.^58^ In another hospital series consisting of 600 patients with Chagas heart disease, 92.2% of patients had an altered electrocardiogram, among which many had more than one electrocardiographic abnormality.^59^

Among the most frequent electrocardiographic abnormalities is sinus bradycardia, which can occur in up to 20% of patients. Extrasystoles can occur in up to 19% of patients, being twice as frequent as in the general population.^60^ Approximately 40% of patients present right bundle branch block (BRD), which is often associated with left anterior fascicular block. In another series, BRD was found in 22.7% of cases and associated with left anterior fascicular block (LAFB) in 13.7%.^58^ First-degree atrioventricular blocks are also common, occurring in approximately 5% of patients. Second- and third-degree blocks are rare, but in hospital series, they can occur in up to 11% of cases.^59^

Other electrocardiographic changes also reflect the presence of heart disease, and low voltage and electric inactive areas can be found in up to 10% of cases.^61^ A 2013 meta-analysis^62^ showed that the most common EKG abnormalities were right bundle branch block (RBBB) and left anterior fascicular block (LAFB) and first-degree A-V block as well as arrhythmias (atrial fibrillation or flutter and ventricular ectopic). An important conclusion was that the prevalence of ECG alterations in children was similar to that in adults and suggests an earlier onset of cardiac disease.^62^

Several electrocardiographic changes are markers of increased risk of death in patients with Chagas disease,^63^ such as the presence of polymorphic and paired ventricular extrasystoles identified in 75% of patients who died in a series of patients with Chagas disease.^64^ In this same series, low voltage and repetitive ventricular arrhythmias occurred in 48.3% of patients who died, correlating significantly with the risk of death (risk ratio of 2.15). It is also considered electrocardiographic predictors of higher risk for sudden death the presence of an electrically inactive zone, left anterior fascicular block, increased dispersion of the QT interval and an increase in the corrected QT.^65^ ^,^ ^66^

The coexistence of pathological Q waves characterises the diffuse and marked impairment of ventricular function and may be related to the presence of an apical aneurysm of the left ventricle. Other severity markers have been proposed, such as the presence of T wave deviation and T wave amplitude variability, changes identified as an independent risk factor for death in these patients.^67^ Recently, an association has been reported between T-wave microalternation and malignant ventricular arrhythmias in Chagas disease.^68^

All evidence converges to a much higher risk of cardiac death in patients with Chagas cardiomyopathy compared with the general population, congruent with the finding that Chagas disease evolves numerous electrical and myocardial abnormalities. The scores developed to stratify this population in relation to the risk of death represent an important achievement for a better understanding of the evolution of the disease and assistance to affected people.^68^ Electrocardiographic markers of prognosis in Chagas heart disease persist as important targets for investigation, and with the continuous evolution of this methodology, new insights will certainly appear.

*Echocardiogram* - Echocardiography is the most common imaging modality used to assess patients with Chagas cardiomyopathy. Cardiac impairment is generally a progressive process that can be classified into stages (A, B, C and D) according to international recommendations adapted to Chagas disease^69^ (Table II). Left ventricular segmental abnormalities are common in Chagas cardiomyopathy at any stage of the disease. These are located mainly at the left ventricular apex and inferior and inferolateral walls, but they may also affect other left ventricular or right ventricular segments.^70^

**TABLE II:** Stages in the development of heart failure due to Chagas disease

Stages^*^	Findings
A	Patients present no symptoms of heart failure and no structural heart disease (normal ECG and chest X-ray)
B1	Asymptomatic patients with ECG changes (arrhythmias or conduction disorders); mild echocardiographic contractile abnormalities with normal global ventricular function can also be present
B2	Patients with decreased left ventricular ejection fraction who have never had any signs or symptoms of heart failure
C	Patients with left ventricular dysfunction and prior or current symptoms of heart failure
D	Patients with symptoms of heart failure at rest, refractory to maximised medical therapy (NYHA IV) that require specialised and intensive interventions

*: I Latin American Guidelines for the diagnosis and treatment of Chagas heart disease: executive summary; ECG: electrocardiogram; NYHA: New York Heart Association.

The prevalence of segmental wall motion abnormalities varies according to the stage of the disease, reaching approximately 50% in patients with left ventricular dilatation and dysfunction.^71^ Diastolic dysfunction is an important hallmark of Chagas disease even in its early phases. In general, left ventricular diastolic and systolic dysfunction coexist, and isolated diastolic dysfunction is uncommon.^72^ ^,^ ^73^ Right ventricular dysfunction is considered a typical feature of Chagas cardiomyopathy. It may be detected early in the disease course, but in general, the clinical manifestations occur late in advanced stages of Chagas cardiomyopathy.^74^ Functional mitral and tricuspid insufficiencies are often associated with severe biventricular global systolic dysfunction (Table II).

Myocardial deformation imaging is a relatively new echocardiographic technique for the quantitative assessment of myocardial contractility.^74^ Strain is a measure of myocardial deformation, defined as the change in length of the myocardium relative to the original length, and it can be measured using speckle tracking echocardiography. Speckle tracking echocardiography allows a more precise and quantitative measurement of regional myocardial function in Chagas cardiomyopathy.^75^ Global longitudinal strain is the most studied method for the detection of subclinical ventricular dysfunction in patients with Chagas cardiomyopathy and is correlated with the amount of myocardial fibrosis.^76^ ^,^ ^77^ Regional strain is of particular interest in Chagas cardiomyopathy, given the frequent segmental myocardial involvement. In addition, strain has also been explored as a potential predictor of adverse outcomes in Chagas cardiomyopathy.^78^ ^,^ ^79^ Other uses of myocardial strain are currently being explored, including the evaluation of mechanical dispersion and risk of arrhythmias.^80^

Therefore, a growing body of evidence suggests that the assessment of cardiac function by echocardiographic myocardial deformation parameters provides incremental information in clinical settings. In the context of Chagas disease, the clinical impact of early myocardial changes assessed by these advanced echocardiographic techniques in predicting disease progression still needs to be well defined.

*Cardiac magnetic resonance* - Cardiac resonance with the delayed enhancement technique after gadolinium infusion plays a fundamental role in risk stratification in patients with chronic Chagas heart disease. In a series of 140 patients with 53.6 ± 11.5 years of life, the occurrence of fibrotic load greater than 12.3 g was associated with higher mortality, ICD therapies or reversed cardiac arrest, regardless of the Rassi score. Interestingly, patients without fibrosis did not experience such events.^81^

Magnetic resonance studies showed late enhancement in 75% of patients with Chagas cardiomyopathy and in 25% of patients with indeterminate forms. The scar was most frequently located on the left ventricular lateral-basal and middle-basal walls.^82^ ^,^ ^83^ Lee-Felker^84^ and collaborators studied scar distribution using delayed enhancement cardiac magnetic resonance in 81 Chagas disease patients residing in the United States (mostly immigrants from El Salvador and Mexico) and demonstrated that the scar was predominantly transmural (56%, 65/117 segments) and mesocardial (30%, 35/117 segments). Endocardial scarring was uncommon (14%, 16/117 segments), and subepicardial scarring was rare (< 1%, 1/117 segments). Interestingly, the distribution of scars on resonance in the Brazilian population was different, ,ore often showing epicardial scars.^85^

Recently, resonance processing software has been used, aiming to quantify the extent of the scar and distribution and mainly to identify the presence of ventricular tachycardia circuits (Fig. 1). In addition, when this information is integrated into the electroanatomical mapping system during ablation, it accelerates procedure faster and has a lower recurrence rate.^86^

**Fig. 1:**
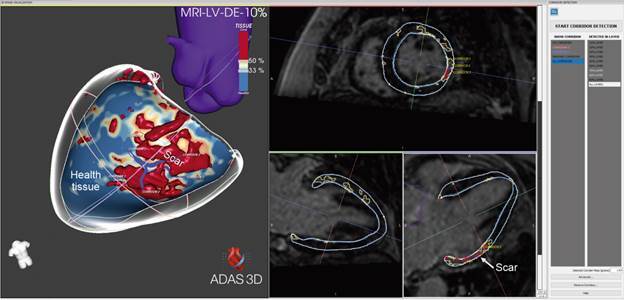
cardiac magnetic resonance with late-gadolinium enhancement with tridimensional reconstruction of the scar in a patient with Chagas cardiomyopathy showing an infero-latero-basal scar with two corridors that are potential circuits for monomorphic ventricular tachycardia (VT).

**Chronic Chagas heart disease and its therapeutic approach: what have large studies taught us about heart failure?**

Despite originally being an infectious illness, Chagas disease must be clinically managed as a chronic disorder, with an important impact on the public health system due to its serious late cardiovascular complications.^87^ The pattern of dilated cardiomyopathy in Chagas disease is chronic myocarditis with a large amount of fibrosis, responsible for significant involvement of the electrical conduction system and formation of re-entrance circuits, causing bradi and tachyarrhythmias and progressive systolic dysfunction, respectively, with segmental wall motion abnormalities usually preceding global impairment.^88^ The common pathophysiology with other aetiologies suggests that elective therapies in usual heart failure with reduced ejection fraction (HFrEF) patients could also be beneficial in Chagas disease.

Abnormalities of systolic and diastolic functions frequently coexist, and right-sided HF symptoms can be more prominent than left-side HF. Isolated pulmonary congestion symptoms and acute lung oedema occur only in the presence of severe left mitral regurgitation, either by mitral tethering due to progressive remodelling of the left ventricle (LV) or by disruption of valve coupling associated with basal inferolateral fibrosis. Right ventricle (RV) dysfunction is usually associated with LV dysfunction in advanced stages of CCC, indicating a poor prognosis.^89^

In addition to an altered electrocardiogram that defines the diagnosis of CCC, echocardiography is essential in HF patients, allowing, as cited below, the assessment of the degree of systolic dysfunction, an analysis of filling pressures and segmental patterns with characteristic aneurysms, and some silent complications, such as the presence of intracavitary thrombus.^90^

In a meta-analysis of 143 studies, Chagas disease was responsible for 36% of all HF cases in Latin America, with a mortality of 1.12-7.18 per 100,000 people per year being reported.^91^ In relation to prognosis, patients with Chagas-HF have a poorer quality of life and higher rates of hospitalisation and mortality than patients with other nonischaemic and ischaemic aetiologies, regardless of younger age, fewer comorbidities and comprehensive use of conventional therapies for HFrEF. They have the highest proportion of hospital admissions for cardiogenic shock and arrhythmia, with lower systolic blood pressure and a higher proportion of right ventricular HF, pacemaker implantation and prevalence of stroke.^92^ ^,^ ^93^ ^,^ ^94^ Chagas disease has become the third most common indication for heart transplantation in South America.^95^ In fact, although Chagas disease is a highly arrhythmogenic cardiomyopathy, modern pharmacological therapy has reduced the risk of sudden death, and pump failure death associated with HF has become the major mode of death in CCC in recent years.^96^ Unfortunately, even today, most of the therapies for Chagas-HF are prescribed based on evidence from expert opinions or clinical trials that included only a small proportion of Chagas disease patients.^90^ ^,^ ^91^

In the chronic phase of the disease, tertiary prevention activities are important to mitigate or reduce morbidity and mortality related to chronic complications while also improving the quality of life of those affected.^87^ The first approach involves dietary guidelines, such as avoiding excessive fluid and salt intake, promoting self-care and adherence to treatment, maintaining regular physical activities, and prohibiting alcohol and tobacco.

Routinely, given that there are no contraindications, a neurohumoral block with beta-blockers, angiotensin-converting enzyme inhibitors (ACEIs) or angiotensin receptor blockers and mineralocorticoid receptor antagonists represents the gold standard treatment for HFrEF. Among studies evaluating pharmacological Chagas-HF treatment, a few randomised clinical trials, including only dozens of patients, and some retrospective studies have demonstrated benefits of ACEIs and beta-blockers, including better survival with beta-blockers.^95^ Blocking the adrenergic response could have specific pathophysiologic relevance in Chagas disease due to the classical autonomic imbalance associated with this cardiomyopathy, with potential development of ventricular arrhythmia.^89^ However, due to the higher degrees of heart block and autonomic nervous system disorders, patients with Chagas-HF receive digitalis and beta-blockers less frequently or do not reach the recommended full dose. Among beta-blockers, carvedilol is the most frequently used medication. A meta-analysis of two trials (69 participants) found a lower proportion of all-cause mortality in the carvedilol group than in the placebo group [RR 0.69; 95% confidence interval (CI) (0.12 - 3.88)], although the evidence was low quality through the GRADE assessment, and there were no conclusive results between carvedilol and placebo in terms of hospital readmissions and quality of life.^97^

In contrast to the lower use of beta-blockers, patients with Chagas-HF more often receive amiodarone due to the higher risk of ventricular malignant arrhythmias. The same phenomenon has been observed with anticoagulants due to a higher frequency of cardioembolic events.^91^ ^,^ ^94^

Digitalis may be added to the initial regimen in patients with refractory symptoms despite standard therapy, especially those with atrial fibrillation with a fast ventricular response. Appropriate monitoring of the serum level and the development of atrioventricular nodal dysfunction is recommended.^89^

Diuretics should be used in clinical evidence of congestion at the lowest possible dosage to obtain a negative balance, thereby avoiding electrolyte and metabolic disorders. They improve the quality of life and relieve congestive symptoms in patients with HF, despite no demonstrated impact on mortality, particularly in CCC.^89^ ^,^ ^91^

Recently, in a *post hoc* subanalysis of the SHIFT trial, patients with Chagas disease demonstrated lower heart rates and improvement in functional class with ivabradine, with a trend towards a reduction in mortality.^98^ However, the indication of ivabradine in Chagas disease patients is limited due to a lower resting heart rate probably caused by electrical conduction system disturbances and higher use of amiodarone.^90^

Evidence for the role of angiotensin receptor neprilysin inhibitors (ARNIs) in CCC is lacking, and only 7.6% of 2,552 Latin American patients with HFrEF randomised in the PARADIGM-HF and ATMOSPHERE trials had Chagas disease. Patients with CCC treated with sacubitril/valsartan, compared with enalapril, had a lower risk of cardiovascular death or HF hospitalisation. This analysis is underpowered and should be interpreted with caution.^99^ A study dedicated to patients with Chagas-HF has been underway since 2019. PARACHUTE-HF is a phase 4, multinational, multicentre, prospective, randomised, controlled study with the objective of demonstrating the superiority of sacubitril/valsartan over enalapril in improving a composite endpoint of cardiovascular death or first HF hospitalisation in Chagas-HF (NCT04023227).

Different devices for cardiac assistance could be used in patients with end-stage Chagas-HF. These could be applied as a bridge to transplantation, a bridge to recovery, or even as destination therapy. Unfortunately, the scenario of social vulnerability and poor access to health services in endemic countries makes this option uncommon, but some successful experiences have been described.^91^ ^,^ ^95^

It has been widely demonstrated that physical exercise promotes several benefits for patients with heart disease in general, mainly of ischaemic and hypertensive aetiologies.^100^ Despite this, there are few studies involving Chagas disease patients. A recent clinical trial, the PEACH study,^101^ showed that the maximum oxygen volume (VO_2_) increased among CCC participants submitted to a cardiac rehabilitation programme. The results of this clinical trial reinforce evidence that physical exercise and other multidisciplinary approaches should be available to Chagas-HF patients.

New insights are necessary to minimise the deep barriers preventing access to treatment of chronic complications, which negatively contribute to the poor prognosis of this heart disease.

**Thromboembolic events**

Systemic embolisms are common manifestations in patients with CCC and can be the first manifestation of the disease even in early stages of the disease without ventricular dysfunction (Stage B2). The most important mechanisms that lead to thromboembolic events are left ventricular systolic dysfunction, arrhythmias, apical aneurysm, and thrombi from cardiac chambers due to blood stasis.^102^ There are few studies estimating the incidence of thromboembolic events in Chagas disease patients. In 1983, a necropsy study showed that systemic or intracardiac thrombi were observed in 44% of 1345 autopsy reports from CCC patients.^103^

Although thromboembolic events are not as frequent as other outcomes, they have great importance due to their high morbidity and mortality, especially concerning cerebrovascular events. A 2014 systematic revision with 4158 patients showed that the risk of stroke was elevated in the group of patients with Chagas disease, reaching 70% higher than that in noninfected patients (RR = 1.70; HF 95%: 1.06 to 2.71).^104^

A prospective cohort with 1043 patients studied the incidence of cardioembolic stroke in CCC, resulting in 3.0% in 5.5 years or 0.56%/year. This cohort formulated the IPEC-FIOCRUZ score with four variables and five points to predict the risk of cardioembolic stroke: systolic dysfunction (two points), presence of apical aneurysm (one point), age over 48 years (one point), and primary change in ventricular repolarisation at electrocardiography (one point). Patients with four-five points would have an incidence as high as 4.4% per year of cardioembolic stroke, in contrast to individuals with an incidence close to zero, with zero-one point.^105^

There were no events in patients using oral anticoagulation with warfarin; however, it added a greater bleeding risk of approximately 2% per year. Thus, oral anticoagulation would be indicated only in subgroups at high risk of cardioembolic events, characterised by patients with four-five points. In two-point patients with a low incidence of stroke (1.2% per year), acetylsalicylic acid or no prophylaxis was recommended. Patients using acetylsalicylic acid did not present haemorrhagic complications; however, this drug is not as effective as warfarin. Patients with an incidence of zero-one points have an incidence close to zero and do not require prophylaxis.^106^ To prevent cardioembolic events in CCC patients, a risk benefit should always be evaluated.^107^ ^,^ ^108^

**Approach to the management of arrhythmias in the current therapeutic context**

The treatment of arrhythmias in Chagas disease, especially sustained ventricular tachycardia (SVT), aims to prevent sudden death. The strategies used are based on the implantation of cardiodefibrillators (ICDs) and on the prevention of the occurrence of arrhythmias using antiarrhythmic drugs and catheter ablation.

Implantable cardioverter defibrillators are frequently used in patients with Chagas heart disease and SVT. However, there are no randomised clinical studies evaluating their role in primary prophylaxis for sudden death in patients with Chagas cardiopathy. The CHAGASICS randomised study is currently underway to evaluate the role of implantable defibrillators in comparison with amiodarone in patients with chronic Chagas heart disease and ventricular dysfunction.^109^

Despite the lack of randomised studies, some observational studies have shown the benefit of these devices. In an investigation of 76 patients with Chagas heart disease and SVT with an LV ejection fraction above 40%, the use of ICD was associated with lower mortality (4.7%) compared with patients who used only amiodarone.^110^ In another investigation, the authors described the follow-up of 90 Chagas patients with SVT who received an ICD implant.^111^ The mean LV ejection fraction was 47 ± 13%, and in an average follow-up of two years, mortality was 34%. Importantly, they observed that patients who presented more than four shocks per month had higher mortality.^111^ In 2018, Carmo AAL et al.^112^ developed a meta-analysis including 115 Chagas patients using amiodarone and 483 with ICD and amiodarone. In that analysis, there was no difference in mortality between patients with or without ICD (9.6% and 9.7%, respectively.^112^

Amiodarone is frequently used in patients with chronic Chagas heart disease even before they have SVT. Patients with more preserved ventricular function seem to benefit from the use of amiodarone;^113^ however, the rate of occurrence of ventricular arrhythmias is still high, and catheter ablation is often necessary.

SVT catheter ablation seeks to homogenise the arrhythmogenic substrate composed of viable myocardial fibres in the middle of the scar. Circuit mapping can be performed during tachycardia; however, due to the complexity of the scars, which can have multiple circuits and SVT morphologies, it is usually performed in sinus rhythm (substrate modification) through the identification of abnormal electrophysiological potentials in the scar area. Electroanatomical mapping is essential for this procedure. An important point in patients with Chagas heart disease is that the substrate is often subepicardial in origin, requiring an epicardial approach in most patients (Fig. 2).

**Fig. 2:**
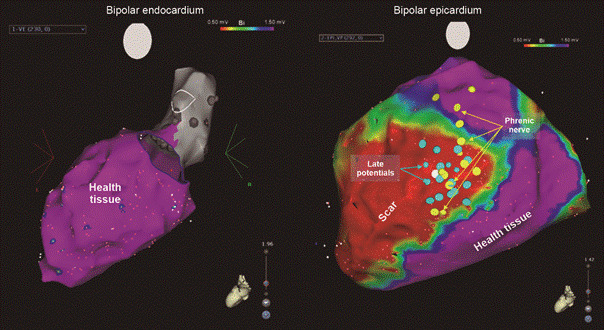
electroanatomical epicardial and endocardial voltage mapping in a patient with chronic Chagas cardiomyopathy. The endocardial voltage map shows no endocardial scar (purple colour), and the epicardial voltage map shows a large epicardial scar (red colour) on the latero-anterior and inferior walls extending from the base of the left ventricle to the apex. There are late potentials that indicate the area of slow conduction on the inferolateral portion of the scar.

In the first series, described in 1999,^114^ the authors performed simultaneous epi- and endocardial mapping in patients with SVT. Ten patients with Chagas disease were studied, in which 18 mappable tachycardias were identified. In this series, at least one epicardial circuit was found in 14 of the 18 mapped SVTs. Epicardial ablation was performed in six patients with interruption of tachycardia.^114^ In another series, the authors^115^ performed endocardial and epicardial mapping in 17 patients with Chagas cardiomyopathy, obtaining immediate success in 14 (83.3%) patients. In a mean follow-up of 318 ± 95 days, 11 of the 14 patients did not experience recurrence of VT; the number of therapies decreased from 4.64 ± 2.71 in the six months before ablation to 0.29 ± 0.61 (p = 0.002) in the same period after.^115^

A randomised study was carried out in 2020^116^ comparing the mapping technique and endocardial versus epicardial ablation in 30 patients with chronic Chagas heart disease and SVT. The procedure was unsuccessful when the ablation was exclusively endocardial in nine (60%) patients. Conversely, only two (13%; p = 0.0215) patients were unsuccessful when the endo- and epicardial approaches were combined. The median area of epicardial scarring was larger (mean of 61.2 cm^2^) than that of the endocardial scar (13.35 cm^2^; p < 0.001). Interestingly, five patients (17%) did not have an endocardial scar; all the investigated patients had an epicardial scar (p = 0.05). In this series, it was also shown that mapping and epicardial ablation were safe, with only two patients presenting a haemopericardium above 80 mL, without major complications related to the procedure. In a median follow-up of 587 days, 60% of patients undergoing exclusive endocardial ablation and 33.3% in the combined endo- and epicardial strategy had recurrence of SVT, with a median time to recurrence of 46.5 days.^116^

Generally, the treatment of ventricular arrhythmias in patients with chronic Chagas heart disease is complex, requiring the use of antiarrhythmic drugs, usually amiodarone, in the presence of significant ventricular dysfunction and an ICD implantation. Even though, when patients present recurrent ICD therapies despite the use of amiodarone, requiring ablation, an epicardial approach in most cases is recommended.

**Cardiac transplantation**

In the past, Chagas disease was considered a contraindication to cardiac transplantation (CT) due to the risk of reactivation of *T. cruzi* infection as a result of immunosuppressive therapy.^117^ Brazil was a pioneer in this procedure, and since the 1980s, CT has become an established alternative for terminal Chagas’s heart disease, with survival rates of 76%, 71% and 46% at six months, five years and 10 years, respectively, and it was better than the cohort of patients undergoing CT due to other aetiologies.^118^ ^,^ ^119^ However, the only national registry that compiled the results of CT in Brazil was carried out in 1999.^120^ Chagas’s disease is the third leading cause of indication for CT in endemic countries, corresponding to 35% of patients undergoing the procedure.^118^ ^,^ ^119^ However, the emigration of Latin American patients with *T. cruzi* to nonendemic countries, such as North America, Europe, Asia and Oceania, has globalised the disease, creating a problem not only epidemiologically but also for transplant teams.^121^ ^,^ ^122^

The indications and contraindications for CT follow the classic criteria, with some peculiarities.^119^ ^,^ ^123^ In general, these patients have a less favourable social and cultural profile, but there does not seem to be a relationship between the socioeconomic situation and the evolution after CT.^124^ ^,^ ^125^ The possibility of megaoesophagus and megacolon should be evaluated, which, depending on the severity, may constitute contraindications.^119^ In Brazil, serology for *T. cruzi* infection in all potential donors and recipients is mandatory and has been recommended in nonendemic countries with potential donors/recipients having a positive epidemiology.^6^ ^,^ ^119^ ^,^ ^123^

Basic maintenance immunosuppressive therapy includes a calcineurin inhibitor (cyclosporine A or tacrolimus) associated with mycophenolate mofetil or mycophenolate sodium or azathioprine and prednisone, with the lowest possible immunosuppressive intensity, as long as rejection does not occur.^119^

The incidence of reactivation after CT varies from 20 to 45% in the first year.^119^ ^,^ ^126^ ^,^ ^127^ Considering the potential morbidity and mortality, the diagnosis and appropriate management of reactivation of Chagas’s disease is extremely important and must be carried out within a structured clinical and laboratory protocol.^6^ ^,^ ^119^ ^,^ ^122^ The diagnosis of reactivation is based on clinical signs and symptoms and/or the presence of parasites in blood, cerebrospinal fluid, bone marrow or tissues.^6^ ^,^ ^119^ ^,^ ^122^

Clinical reactivation has cardiac and extracardiac manifestations, including myocarditis, skin lesions, fever, bone marrow involvement and neurological manifestations.^119^ ^,^ ^126^ ^,^ ^128^ Reactivation myocarditis can be mistakenly diagnosed as graft rejection and treated with an intensified immunosuppressive treatment, which will aggravate reactivation.^119^ ^,^ ^127^ The differential diagnosis between rejection and reactivation myocarditis is complex.^119^ ^,^ ^127^ In the presence of an inflammatory infiltrate, amastigote nests and/or positive polymerase chain reaction (PCR) for *T. cruzi* in the myocardium, we can say that there is reactivation, but it is not possible to safely exclude the associated graft rejection.

Serological tests are useful only in potential donors and recipients.^6^ ^,^ ^119^ They have no role in the diagnosis of reactivation. Traditionally, laboratory monitoring has utilised parasitological methods (direct research of *T. cruzi* in blood and blood cultures) and serial histological examinations of endomyocardial biopsies in search of *T. cruzi* amastigote tests with low sensitivity.^6^ ^,^ ^119^ In recent years, several studies have demonstrated the PCR test value in peripheral blood and fragments of endomyocardial biopsy (EMB) in detecting early reactivation before the appearance of symptoms and/or graft dysfunction.^119^ ^,^ ^129^ ^,^ ^130^

In the presence of signs/symptoms and/or the identification of the parasite in the blood, cerebrospinal fluid or tissue, it is recommended to start aetiological treatment immediately. This condition, when treated early and properly, has a good prognosis. Benznidazole is the recommended first-line treatment^6^ ^,^ ^119^ delivered at 5 mg/kg/day for 60 days, with the daily dose being divided two or three times. Nifurtimox is not available in Brazil. There is no evidence to support a prophylactic anti-*T. cruzi* treatment strategy for reactivation. A patient may have more than one reactivation episode after treatment. Therefore, it is necessary to maintain the monitoring of reactivation even after anti-*T. cruzi* treatment.^6^ ^,^ ^119^ ^,^ ^131^ The aetiological treatment of patients with a positive PCR in EMB and without clinical manifestations of reactivation remains to be established.^127^

**Perspectives and challenges in cardiac transplantation**

The globalisation of Chagas disease requires attention and knowledge from transplant teams in nonendemic countries, and failures in the diagnosis and treatment of reactivation can have fatal consequences. With the incorporation of PCR techniques, the reactivation concept should be revised. The differential diagnosis between rejection and reactivation remains a challenge and requires further study. Multicentre studies comparing different immunosuppression strategies are desirable, as well as a national registry to assess management, patient care and the results of the procedure.

**Acute Chagas’s heart disease: a new and poorly known challenge**

The annual incidence of acute cases of Chagas disease due to vector transmission has been decreasing in Latin America and Brazil.^6^ Currently, Chagas disease presents itself in the acute form in countries of the American continent and in several states of Brazil, with the increase in new cases attributed to oral transmission due to lack of hygiene in food processing.^132^ In fact, the disease shows a higher incidence of oral transmission in the Brazilian Amazon, mainly in the states of the Northern region of Brazil, with a clinical-epidemiological scenario that differs from the classic vector transmission recorded in the Southeastern and Central West regions, which are historically endemic in the country.^133^ ^,^ ^134^

The common form of presentation is observed in outbreaks of familial microepidemics and, less frequently, in isolated cases, but it is related to the lack of food hygiene.^135^ Oral transmission by food has drawn the attention of the scientific community to better understand this route of transmission and the way food is contaminated, especially açai, an Amazonian fruit that is frequently involved in this chain of transmission.^136^

Certain gaps need to be better defined, such as the parasite’s entrance doors in the human organism using the oral route and the inoculum’s relationship with morbidity, mortality and clinical evolution. In this sense, studies are scarce and have been well documented in experimental models, showing different doors of entry through the oral route that are related to the disease evolution.^137^ Symptomatic patients may have prolonged febrile symptoms and nonspecific symptoms characteristic of infectious processes that generate diagnostic confusion with typhoid fever, malaria and kalaazar.^135^ ^,^ ^138^

The acute phase due to oral transmission also differs from the vector-transmitted acute phase from the clinical perspective. Oral infection has a greater number of symptoms, and fever is more present and prolonged, with greater morbidity, suggesting that it is related to the inoculum.^139^ Acute infection suggests greater morbidity and lethality in males, as evidenced in a series of acute cases.^140^ ^,^ ^141^

The lack of a definition of clinical prognostic and evolutionary markers of acute disease represents another problem, since the registered reports are only descriptive studies of case series.^142^ ^,^ ^143^ The fatality of some symptomatic cases is observed with clinical signs of severe heart failure and/or septic shock. In contrast, groups of poorly symptomatic patients may present slight changes in the electrocardiogram already showing early myocardial injury.^133^ ^,^ ^144^ Moreover, in a subgroup of poorly symptomatic individuals, most of the series presented an absence of nocturnal sleep decline in blood pressure, suggesting an impairment of the autonomic nervous system already in the initial phase, corroborating studies describing dysautonomy in the acute phase of infection.^145^ The clinical significance of the involvement of the autonomic nervous system is not well established in Chagas’s disease as a prognostic marker of progression to cardiac sequelae, requiring longitudinal studies to answer this question.^146^

Concerning the diagnostic methods for detecting the parasite in the bloodstream, the scarcity of sensitive, rapid and easily accessible tests for the diagnosis of the acute phase of Chagas disease has been perpetuated for decades without technological advances, which is still a great challenge in rural locations located far from urban centres.

There is a pressing need for studies to better define the strains and parasitic load in endemic regions of the Amazon, with cutting-edge technological investments to flatten the annual seasonality curve to minimise the incidence of acute cases.^147^ Scientific evidence related to treatment with antiparasitic agents administered in the acute phase is still scarce. Reports of acute and fatal cases in the Amazon region have evolved with septic shock and ventricular dysfunction, some under aetiologic monotherapy treatment since, to date, only benznidazole has been available in Brazil, without robust studies pointing to a change in the prognosis of patients treated in the initial stage of infection.^143^ ^,^ ^144^

In the case of pregnant women with acute Chagas’s disease due to oral transmission, there is no consensus on the gestational period in which it will be safe to prescribe aetiological treatment due to the absence of evidence confirming foetal teratogenicity by antiparasitic agents, implying ethical aspects in medical decisions.^6^ ^,^ ^148^

Another aspect to consider is that we are facing an acute infectious disease with chronic evolution of an inflammatory character in the acute phase, which suggests an alteration of coagulation factors, with thrombus and haemorrhage previously described.^143^ ^,^ ^149^ In the pandemic scenario caused by Coronavirus disease 2019 (COVID-19), we warn of the possibility of coinfection at a time when there is no scientific evidence of the evolution of cases of coinfection of acute Chagas disease with other infectious diseases.

Investment in public food hygiene policies is necessary to minimise the occurrence of acute infection by oral transmission in the affected locations. As it is a chronic infectious disease, without robust evidence of a cure and a lack of randomised studies, prevention is currently the best alternative.

A series of challenges still need to be overcome and revolve around studies of genotyping and immunopathogenicity of the parasite in endemic areas. New therapies, unravelling clinical and inflammatory markers that cause sequelae, and the development of quick, more accessible and specific alternative methodologies for diagnosing parasitic infection are needed. However, the most fundamental necessity is the investment in new therapeutic options for aetiological treatment of the acute phase, which have evidence of efficacy and safety for eliminating the parasite with fewer adverse events.

**Ageing and comorbidities: a new reality for patients with chronic Chagas heart disease in Brazil**

The change in the epidemiological profile of Chagas’s disease has transformed the old profile of rural youth patients into older patients living on the outskirts of large cities experiencing an era of chronic degenerative diseases. The socioeconomic status of these patients, per se, already predisposes them to the appearance of other conditions.^150^ The impact of comorbidities among patients with chronic Chagas heart disease requires well-defined studies. Knowledge from other areas is usually extrapolated, indicating that, in general, good clinical practice should be followed with the same therapeutic guidelines. It is necessary to contemplate periodic monitoring and, when necessary, to consult with specialists. Pharmacological/nonpharmacological guidance should be promptly instituted, following what is recommended by specific current guidelines.^151^ ^,^ ^152^ ^,^ ^153^

High blood pressure is the most frequent cardiac comorbiditie associated with CCC.^154^ ^,^ ^155^ The natural course of untreated systemic arterial hypertension is the development of heart failure (HF); therefore, it is necessary to diagnose this condition early and provide aggressive treatment to reduce blood pressure levels to target values.

Coronary artery disease (CAD) is a challenge in patients in older age groups, since CCC patients can report chest pain, mimicking CAD, despite the common finding of normal subepicardial coronaries after angiographic investigation. Several theories attempt to explain this anginoid syndrome, which may be related to dysfunction in the microcirculation, abnormal vascular reactivity and alteration of the endothelium. The presence of sympathetic denervation has also been considered, which can lead to loss of vasomotor tone with a consequent alteration of the coronary flow, potentially generating myocardial ischaemia.^156^ ^,^ ^157^ In these cases, myocardial perfusion scintigraphy presents regional motility changes as well as transient and/or permanent perfusion defects, which may suggest obstructive coronary disease.^158^ These aspects must be taken into account in patients with CCC and suspected CAD, and cardiac catheterisation is often indispensable.

Among noncardiological comorbidities, diabetes mellitus (DM2) and chronic obstructive pulmonary disease (COPD) are prominent. It is estimated that approximately one-quarter to one-fifth of elderly patients with moderate to severe HF have COPD. The differential diagnosis of the origin of dyspnoea is essential to define the most appropriate management.^159^ The use of beta-blockers in patients with CCC who are undergoing COPD and HF must be maintained, as it reduces hospitalisations and mortality due to HF. In this scenario, bisoprolol has shown a greater safety and benefit than carvedilol and metoprolol.^160^

Diabetes mellitus is a comorbidity that has been growing in CCC patients due to the ageing of this population and, when associated with HF, increased mortality.^161^ Metformin, which is associated with lifestyle changes, remains the initial therapy of choice for most of these patients due to its proven benefit in reducing blood glucose, easy access, low cost and, above all, relationship with reduced cardiovascular mortality. In patients who require a combination with other drugs to control glycaemia, one should opt, whenever possible, for those that have been shown to have a positive impact in reducing cardiovascular morbidity and mortality. In this sense, ISGLT2 is noteworthy, which, despite a high cost, has shown a reduction in hospitalisations due to HF decompensation and mortality.^162^ ^,^ ^163^

**Challenges and public policies for the diagnosis, treatment and monitoring of patients with chronic Chagas heart disease**

It is estimated that there are 8 to 10 million people infected with *T. cruzi* worldwide. Of this total, 2 to 3 million refer to the estimated prevalence of the disease in Brazil. Considering the percentage of people who progress to the cardiac form of Chagas disease, it is estimated that there are currently between 600 and 900 thousand people with chronic Chagas heart disease in the country, the majority of which are in older age groups, a population with a higher frequency of comorbidities.^7^

Considering the actuarial charts available, we can expect the existence of people with chronic heart disease for thirty or forty more years in Brazil or even longer if we consider the population of the Amazon region that is being infected orally at present and may develop chronic heart disease in the future. It is possible that we are now perceiving only the tip of the iceberg with regard to the future consequences of changing the current epidemiological profile of the disease in the Amazon region. Additionally, the lack of a definition of clinical prognostic markers and acute Chagas disease evolution by oral transmission still need to be well studied.

Considering the important prevalence of the disease and the human and social losses it causes, the prevention and management of Chagas heart disease remains a challenge to science and public policies in endemic countries.^6^ ^,^ ^8^

In terms of public health management, it is important to prioritise the implementation of strategies to face situations that have not yet been overcome, even after more than a century of the discovery of the disease. Early detection of Chagas disease and its clinical-evolutionary characterisation are feasible by means of the diagnostic arsenal currently available, but its universalisation is still far from being possible, both in endemic rural regions and in urban centres, due to the lack of knowledge of the disease by health professionals and also due to problems of patient access to different levels of public health care.^7^

As the management of heart disease requires continuity of care, it is necessary to seek the best solutions for its diagnosis, staging, treatment and, above all, to ensure patient compliance with treatment. To achieve these goals, it is essential to invest in the education of health teams, and in a country of continental dimensions such as Brazil, the training and updating of professionals can be achieved through the use of distance education, which has gained significant momentum in recent years, fostered by more universal access to mobile technology and the internet. At universities, technological infrastructure and expertise create and offer, in the distance learning mode, refresher courses on the diagnosis and management of Chagas disease for healthcare professionals. In a more daring concept, we can think of distance education as part of a broader innovation process, involving information, education, monitoring and assistance to citizens with the disease and their families, through the use of telemedicine and tele-education as part of the effort so that information and care reach everyone affected.

Other concrete challenges involve making decisions and setting priorities, which are important to overcome. Funding institutions and researchers are also part of this important process of searching for solutions to reduce suffering and improve the quality of life of patients with Chagas heart disease. There is an urgent need to improve the therapeutic arsenal, both for aetiological treatment and for the management of heart disease itself. Maintaining continued investments in research is essential, since a better knowledge of the pathogenesis of CCC may allow the prevention and treatment of cardiac fibrosis, a major clinical challenge to be overcome. In addition to the search for new chemotherapeutic drugs, investment in a possible vaccine is an important aspect of research, in view of the latest biotechnology and genetic engineering techniques applied to the development of vaccines.

## References

[1] Chagas C (1911). Moléstia de Carlos Chagas ou tireoidite parasitada. Nova doença humana transmitida pelo Barbeiro (Conorhinus megistus). II Conferência à Academia Nacional de Medicina em agosto de 1911. Tipografia Leuszinger.

[2] Chagas C (1916). Processos patojenicos da tripanozomiase americana. Mem Inst Oswaldo Cruz.

[3] Chagas C, Villela E (1922). Cardiac form of American Trypanosomiasis. Mem Inst Oswaldo Cruz.

[4] Chagas E (1930). Forma cardiaca da Trypanosomiase Americana. Memórias do Instituto Oswaldo Cruz.

[5] Dias JCP, Schofield CJ (1999). The evolution of Chagas disease (American Trypanosomiasis) control after 90 years since Carlos Chagas discovery. Mem Inst Oswaldo Cruz.

[6] Dias JC, Ramos AN, Gontijo ED, Luquetti A, Shikanai-Yasuda MA, Coura JR (2016). 2nd Brazilian Consensus on Chagas Disease, 2015. Rev Soc Bras Med Trop.

[7] Dias JCP (2017). Facing Chagas disease. Rev Soc Bras Med Trop.

[8] de Andrade JP, Marin JA, de Paola AAV, Vilas-Boas F, Oliveira GMM, Bacal F (2011). I Diretriz Latino-Americana para o diagnóstico e tratamento da cardiopatia chagásica resumo executivo. Arq Bras Cardiol.

[9] Ribeiro AL, Nunes MP, Teixeira MM, Rocha MO (2012). Diagnosis and management of Chagas disease and cardiomyopathy. Nat Rev Cardiol.

[10] Nunes MC, Dones W, Morillo CA, Encina JJ, Ribeiro AL (2013). Council on Chagas disease of the Interamerican Society of C Chagas disease: an overview of clinical and epidemiological aspects. J Am Coll Cardiol.

[11] Higuchi ML, Benvenuti LA, Reis MM, Metzger M (2003). Pathophysiology of the heart in Chagas' disease current status and new developments. Cardiovasc Res.

[12] Lannes-Vieira J, Pereira IR, Vinagre NF, Arnez LE (2011). TNF-aand TNFR in Chagas disease from protective immunity to pathogenesis of chronic cardiomyopathy. Adv Exp Med Biol.

[13] Tanowitz HB, Scherer PE, Mota MM, Figueiredo LM (2017). Adipose tissue a safe haven for parasites?. Trends Parasitol.

[14] Nagajyothi JF, Weiss LM (2019). Advances in understanding the role of adipose tissue and mitochondrial oxidative stress in Trypanosoma cruzi infection. F1000Res.

[15] Prata A (2001). Clinical and epidemiological aspects of Chagas disease. Lancet Infect Dis.

[16] Rassi A, Rassi A, Marin-Neto JA (2010). Chagas disease. Lancet.

[17] Marin-Neto JA, Cunha-Neto E, Maciel BC, Simoes MV (2007). Pathogenesis of chronic Chagas heart disease. Circulation.

[18] Dutra WO, Rocha MOC, Teixeira MM (2005). The clinical immunology of human Chagas disease. Trends Parasitol.

[19] Parada H, Carrasco HA, Añez N, Fuenmayor C, Inglessis I (1997). Cardiac involvement is a constant finding in acute Chagas'disease a clinical, parasitological and histopathological study. Int J Cardiol.

[20] Nogueira PM, Ribeiro K, Silveira AC, Campos JH, Martins-Filho OA, Bela SR (2015). Vesicles from different Trypanosoma cruzi strains trigger differential innate and chronic immune responses. J Extracell Vesicles.

[21] Sherbuk JE, Okamoto EE, Marks MA, Fortuny E, Clark EH, Galdos-Cardenas G (2015). Biomarkers and mortality in severe Chagas cardiomyopathy. Glob Heart.

[22] Bocchi EA, Bestetti RB, Scanavacca MI, Cunha E, Issa VS (2017). Chronic Chagas heart disease management from etiology to cardiomyopathy treatment. J Am Coll Cardiol.

[23] Silva JS, Vespa GN, Cardoso MA, Aliberti JC, Cunha FQ (1995). Tumor necrosis factor alpha mediates resistance to Trypanosoma cruzi infection in mice by inducing nitric oxide production in infected gamma interferon-activated macrophages. Infect Immun.

[24] Bilate AM, Salemi VM, Ramires FJ, de Brito T, Russo M, Fonseca SG (2007). TNF blockade aggravates experimental chronic Chagas disease cardiomyopathy. Microbes Infect.

[25] Pereira IR, Vilar-Pereira G, Silva AA, Moreira OC, Britto C, Sarmento ED (2014). Tumor necrosis factor is a therapeutic target for immunological unbalance and cardiac abnormalities in chronic experimental Chagas' heart disease. Mediators Inflamm.

[26] Gibaldi D, Vilar-Pereira G, Pereira IR, Silva AA, Barrios LC, Ramos IP (2020). CCL3/macrophage inflammatory protein-1a is dually involved in parasite persistence and induction of a TNF- and IFN -enriched inflammatory milieu in Trypanosoma cruzi- induced chronic cardiomyopathy. J Front Immunol.

[27] Ferreira RC, Ianni BM, Abel LCJ, Buck P, Mady C, Kalil J (2003). Increased plasma levels of tumor necrosis factor-a in asymptomatic/"indeterminate" and Chagas disease cardiomyopathy patients. Mem Inst Oswaldo Cruz.

[28] Talvani A, Rocha MOC, Cogan J, Maewal P, de Lemos J, Ribeiro ALP (2004). Brain natriuretic peptide and left ventricular dysfunction in Chagas cardiomyopathy. Mem Inst Oswaldo Cruz.

[29] Cruz JS, Machado FS, Ropert C, Roman-Campos D (2017). Molecular mechanisms of cardiac electromechanical remodeling during Chagas disease role of TNF and TGF-ß. Trends Cardiovasc. Med.

[30] Curvo EOV, Ferreira RR, Madeira FS, Alves GF, Chambela MC, Mendes VG (2018). Correlation of transforming growth factor-ß1 and tumour necrosis factor levels with left ventricular function in Chagas disease. Mem Inst Oswaldo Cruz.

[31] Moreira MCV, Heringer-Walther S, Wessel N, Ventura TM, Wang Y, Schultheiss HP (2008). Prognostic value of natriuretic peptides in Chagas'disease a 3-year follow-up investigation. Cardiology.

[32] Talvani A, Rocha MOC, Barcelos LS, Gomes YM, Ribeiro AL, Teixeira MM (2004). Elevated concentrations of CCL2 and tumor necrosis factor-alpha in chagasic cardiomyopathy. Clin Infect Dis.

[33] Oliveira BMR, Botoni FA, Ribeiro ALP, Pinto AS, Reis AM, Nunes MCP (2009). Correlation between BNP levels and Doppler echocardiographic parameters of left ventricle filling pressure in patients with Chagasic cardiomyopathy. Echocardiography.

[34] Ribeiro ALP, Teixeira MM, Reis AM, Talvani A, Perez AA, Barros MVL (2006). Brain natriuretic peptide based strategy to detect left ventricular dysfunction in Chagas disease a comparison with the conventional approach. Int J Cardiol.

[35] Gomes JAS, Araújo FF, Vitelli-Avelar DM, Sathler-Avelar R, Lage PS, Wendling APB (2018). Expression, impaired regulatory cytokine microenvironment interfaced with Anti-Trypanosoma cruzi IgG reactivity in cardiac Chagas disease patients. Front Microbiol.

[36] Keating SM, Deng X, Fernandes F, Cunha-Neto E, Ribeiro AL, Adesina B (2015). Inflammatory and cardiac biomarkers are differentially expressed in clinical stages of Chagas disease. Int J Cardiol.

[37] Okamoto EE, Sherbuk JE, Clark EH, Marks MA, Gandarilla O, Galdos-Cardenas G (2014). Biomarkers in Trypanosoma cruzi-infected and uninfected individuals with varying severity of cardiomyopathy in Santa Cruz, Bolivia. PLoS Negl Trop Dis.

[38] Medeiros NI, Gomes JAS, Fiuza JA, Sousa GR, Almeida EF, Novaes RO (2019). MMP-2 and MMP-9 plasma levels are potential biomarkers for indeterminate and cardiac clinical forms progression in chronic Chagas disease. Sci Rep.

[39] Bautista-López NL, Morillo CA, López-Jaramillo P, Quiroz R, Luengas C, Silva SY (2013). Matrix metalloproteinases 2 and 9 as diagnostic markers in the progression to Chagas cardiomyopathy. Am Heart J.

[40] Tobar IB, Parra F, Pérez CN, Rodríguez-Bonfante C, Useche F, Bonfante-Cabarcas R (2011). Prevalence of Trypanosoma cruzi antibodies and inflammatory markers in uncompensated heart failure. Rev Soc Bras Med Trop.

[41] Guedes PMM, Gutierrez FRS, Silva GK, Dellalibera-Joviliano R, Rodrigues GJ, Bendhack LM (2012). Deficient regulatory T cell activity and low frequency of IL-17-producing T cells correlate with the extent of cardiomyopathy in human Chagas'disease. PLoS Negl Trop Dis.

[42] Sousa GR, Gomes JAS, Fares RCG, Damásio MPS, Chaves AT, Ferreira KS (2014). Plasma cytokine expression is associated with cardiac morbidity in Chagas disease. PLoS One.

[43] Gómez-Olarte S, Bolaños NI, Echeverry M, Rodrígues AN, Cuéllar A, Puerta CJ, et al intermediate monocytes and cytokine production associated with severe forms of Chagas disease (2019). Front. Immunol.

[44] Alba-Alvarado MD, Salazar-Schettino PM, Jiménez-Álvarez L, Cabrera-Bravo M, García-Sancho C, Zenteno E (2018). Th-17 cytokines are associated with severity of Trypanosoma cruzi chronic infection in pediatric patients from endemic areas of Mexico. Acta Trop.

[45] Bestetti RB, Dellalibera-Joviliano R, Lopes GS, Faria-Jr M, Furlan-Daniel R, Lopes KC (2019). Determination of the Th1, Th2, Th17, and Treg cytokine profile in patients with chronic Chagas heart disease and systemic arterial hypertension. Heart Vessels.

[46] Ferreira LRP, Ferreira FM, Nakaya HI, Deng X, Cândido DS, Oliveira LC (2017). Blood gene signatures of Chagas cardiomyopathy with or without ventricular dysfunction. J Infect Dis.

[47] Wen JJ, Zago MP, Nuñez S, Gupta S, Burgos FN, Garg NJ (2012). Serum proteomic signature of human chagasic patients for the identification of novel potential protein biomarkers of disease. Mol Cell Proteomics.

[48] Nonaka CKV, Macêdo CT, Cavalcante BRR, Alcântara AC, Silva DN, Bezerra MR (2019). Circulating miRNAs as potential biomarkers associated with cardiac remodeling and fibrosis in Chagas disease cardiomyopathy. Int J Mol Sci.

[49] Nogueira LG, Santos RHB, Ianni BM, Fiorelli AI, Mairena EC, Benvenuti LA (2012). Myocardial chemo kine expression and intensity of myocarditis in Chagas cardiomyopathy are controlled by polymorphisms in CXCL9 and CXCL10. PLoS Negl Trop Dis.

[50] Passos LSA, Villani FNA, Magalhães LMD, Gollob KJ, Antonelli LRV, Nunes MCP (2016). Blocking of CD1d decreases Trypanosoma cruzi-induced activation of CD4-CD8- T Cells and modulates the inflammatory response in patients with Chagas heart disease. J Infect Dis.

[51] Damasio MPS, Rocha MOC, Sousa GR, Ferreira KS, Fares-Gusmão RCG, Medeiros NI (2019). PD1 and PDL1 molecules control suppressor activity of regulatory T cells in chronic Chagas cardiomyopathy patients. Immunol.

[52] Ribeiro AL, Nunes MP, Teixeira MM, Rocha MO (2012). Diagnosis and management of Chagas disease and cardiomyopathy. Nat Rev Cardiol.

[53] Nunes MCP, Dones W, Morillo CA, Encina JJ, Ribeiro AL, Council on Chagas Disease of the Interamerican Society of Cardiology (2013). Chagas disease an overview of clinical and epidemiological aspects. J Am Coll Cardiol.

[54] Prata A (2001). Clinical and epidemiological aspects of Chagas disease. Lancet Infect Dis.

[55] Rassi A, Rassi A, Marin-Neto JA (2010). Chagas disease. Lancet.

[56] Marin-Neto JA, Cunha-Neto E, Maciel BC, Simoes MV (2007). Pathogenesis of chronic Chagas heart disease. Circulation.

[57] Bern C (2015). Chagas' disease. N Engl J Med.

[58] Marcolino MS, Palhares DM, Ferreira LR, Ribeiro AL (2015). Electrocardiogram and Chagas disease a large population database of primary care patients. Global Heart.

[59] Arteaga-Fernández E, Barretto AC, Mady C, Ianni BM, Bellotti G, Pileggi F (1985). [The electrocardiogram in patients with positive serological reactions for Chagas' disease. Study of 600 cases]. Arq Bras Cardiol.

[60] Rocha ALL, Lombardi F, Rocha MOC, Barros MVL, Val Barros VC, Reis AM (2006). Chronotropic incompetence and abnormal autonomic modulation in ambulatory Chagas disease patients. Ann Noninvasive Electrocardiol.

[61] Espinosa R, Carrasco HA, Belandria F, Fuenmayor AM, Molina C, Gonzalez R (1985). Life expectancy analysis in patients with Chagas'disease prognosis after one decade (1973-1983). Int J Cardiol.

[62] Rojas LZ, Glisic M, Pletsch-Borba L, Echeverría LE, Bramer WM, Bano A (2018). Electrocardiographic abnormalities in Chagas disease in the general population a systematic review and meta-analysis. PLoS Negl Trop Dis.

[63] Maguire JH, Hoff R, Sherlock I, Guimaraes AC, Sleigh AC, Ramos NB (1987). Cardiac morbidity and mortality due to Chagas'disease prospective electrocardiographic study of a Brazilian community. Circulation.

[64] Xavier SS, Sousa AS, do Brasil PEAA, Gabriel FG, Holanda MT, Hasslocher-Moreno A (2005). Incidência e preditores de morte súbita na cardiopatia chagásica crônica com função sistólica preservada. Rev SOCERJ.

[65] Salles G, Xavier SS, Cardoso CR, Hasslocher-Moreno A (2003). Prognostic value of QT interval parameters for mortality risk stratification in Chagas' disease results of a long-term follow-up study. Circulation.

[66] Zampa HB, Moreira DAR, Ferreira CAB, Souza CR, Menezes CC, Hirata HS (2014). Valor do ângulo Qrs-T na predição de indução de taquiarritmias ventriculares em pacientes chagásicos. Arq Bras Cardiol.

[67] Almeida BCS, Carmo AAL, Barbosa MPT, Silva JLP, Ribeiro ALP (2018). Association between microvolt T-wave alternans and malignant ventricular arrhythmias in Chagas disease. Arq Bras Cardiol.

[68] Rassi A, Rassi A, Little WC, Xavier SS, Rassi SG, Rassi AG (2006). Development and validation of a risk score for predicting death in Chagas' heart disease. N Engl J Med.

[69] Nunes MC, Barbosa MM, Ribeiro AL, Colosimo EA, Rocha MO (2009). Left atrial volume provides independent prognostic value in patients with Chagas cardiomyopathy. J Am Soc Echocardiogr.

[70] Nunes MC, Kreuser LJ, Ribeiro AL, Sousa GR, Costa HS, Botoni FA (2015). Prevalence and risk factors of embolic cerebrovascular events associated with Chagas heart disease. Global Heart.

[71] Nunes MC, Barbosa MM, Rocha ES, Rocha MO (2005). Function of the left atrium in Chagas' cardiomyopathy. Arq Bras Cardiol.

[72] Rocha MOC, Nunes MCP, Ribeiro AL (2009). Morbidity and prognostic factors in chronic chagasic cardiopathy. Mem Inst Oswaldo Cruz.

[73] Andrade JP, Marin JA, Paola AA, Vilas-Boas F, Oliveira GM, Bacal F (2011). I Latin American guidelines for the diagnosis and treatment of Chagas' heart disease executive summary. Arq Bras Cardiol.

[74] Acquatella H, Asch FM, Barbosa MM, Barros M, Bern C, Cavalcante JL (2018). Recommendations for multimodality cardiac imaging in patients with Chagas disease a report from the american society of echocardiography in collaboration with the interamerican association of echocardiography (ecosiac) and the cardiovascular imaging department of the brazilian society of cardiology (dic-sbc). J Am Soc Echocardiogr.

[75] Acquatella H (2007). Echocardiography in Chagas heart disease. Circulation.

[76] Nunes MP, Colosimo EA, Reis RC, Barbosa MM, da Silva JL, Barbosa F (2012). Different prognostic impact of the tissue doppler-derived e/e' ratio on mortality in Chagas cardiomyopathy patients with heart failure. J Heart Lung Transplant.

[77] Nunes MC, Carmo AA, Rocha MO, Ribeiro AL (2012). Mortality prediction in Chagas heart disease. Expert Rev Cardiovasc Ther.

[78] Geyer H, Caracciolo G, Abe H, Wilansky S, Carerj S, Gentile F (2010). Assessment of myocardial mechanics using speckle tracking echocardiography fundamentals and clinical applications. J Am Soc Echocardiogr.

[79] Hasselberg NE, Haugaa KH, Bernard A, Ribe MP, Kongsgaard E, Donal E (2016). Left ventricular markers of mortality and ventricular arrhythmias in heart failure patients with cardiac resynchronization therapy. Eur Heart J Cardiovasc Imaging.

[80] Barbosa MM, Costa Rocha MO, Vidigal DF, Carneiro RCB, Araujo RD, Palma MC (2014). Early detection of left ventricular contractility abnormalities by two-dimensional speckle tracking strain in Chagas' disease. Echocardiography.

[81] Senra T, Ianni BM, Costa ACP, Mady C, Martinelli-Filho M, Kalil-Filho R (2018). Long-term prognostic value of myocardial fibrosis in patients with Chagas cardiomyopathy. J Am Coll Cardiol.

[82] Torreao JA, Ianni BM, Mady C, Naia E, Rassi CH, Nomura C (2015). Myocardial tissue characterization in Chagas' heart disease by cardiovascular magnetic resonance. J Cardiovasc Magn Reson.

[83] Duran-Crane A, Rojas CA, Cooper LT, Medina HM (2020). Cardiac magnetic resonance imaging in Chagas' disease a parallel with electrophysiologic studies. Int J Cardiovasc Imaging.

[84] Lee-Felker SA, Thomas M, Felker ER, Traina M, Salih M, Hernandez S (2016). Value of cardiac MRI for evaluation of chronic Chagas disease cardiomyopathy. Clin Radiol.

[85] Rochitte CE, Nacif MS, de Oliveira Jr AC.Siqueira-Batista R.Marchiori E.Uellendahl M (2007). Cardiac magnetic resonance in Chagas' disease. Artificial Organs.

[86] Soto-Iglesias D, Penela D, Jauregui B, Acosta J, Fernandez-Armenta J, Linhart M (2020). Cardiac magnetic resonance-guided ventricular tachycardia substrate ablation. JACC Clin Electrophysiol.

[87] Echeverría LE, Marcus R, Novick G, Sosa-Estani S, Ralston K, Zaidel EJ (2020). WHF IASC roadmap on Chagas disease. Glob Heart.

[88] Marin-Neto JA, Cunha-Neto E, Maciel BC, Simões MV (2007). Pathogenesis of chronic Chagas heart disease. Circulation.

[89] Nunes MCP, Beaton A, Acquatella H, Bern C, American Heart Association Rheumatic Fever.Endocarditis and Kawasaki Disease Committee of the Council on Cardiovascular Disease in the YoungCouncil on Cardiovascular and Stroke NursingStroke Council (2018). Chagas cardiomyopathy an update of current clinical knowledge and management: a scientific statement from the American Heart Association. Circulation.

[90] Martinez F, Perna E, Perrone SUV, Aliprandi AS (2019). Chagas disease and heart failure an expanding issue worldwide. Eur Cardiol.

[91] Vilas Boas LG, Bestetti RB, Otaviano AP, Cardinalli-Neto A, Nogueira PR (2013). Outcome of Chagas cardiomyopathy in comparison to ischemic cardiomyopathy. Int J Cardiol.

[92] Freitas HF, Chizzola PR, Paes AT, Lima AC, Mansur AJ (2005). Risk stratification in a Brazilian hospital-based cohort of 1220 outpatients with heart failure role of Chagas' heart disease. Int J Cardiol.

[93] Terhoch CB, Moreira HF, Ayub-Ferreira SM, Conceição-Souza GE, Salemi VMC, Chizzola PR (2018). Clinical findings and prognosis of patients hospitalized for acute decompensated heart failure analysis of the influence of Chagas etiology and ventricular function. PLoS Negl Trop Dis.

[94] Bocchi EA, Bestetti RB, Scanavacca MI, Cunha E, Issa VS (2017). Chronic Chagas heart disease management from etiology to cardiomyopathy treatment. J Am Coll Cardiol.

[95] Ayub-Ferreira SM, Mangini S, Issa VS, Cruz FD, Bacal F, Guimarães GV (2013). Mode of death on Chagas heart disease comparison with other etiologies. A subanalysis of the REMADHE prospective trial. PLoS Negl Trop Dis.

[96] Shen L, Ramires F, Martinez F, Bodanese LC, Echeverría LE, PARADIGM-HF and ATMOSPHERE Investigators and Committees (2017). Contemporary characteristics and outcomes in Chagasic heart failure compared with other nonischemic and ischemic cardiomyopathy. Circ Heart Fail.

[97] Marti-Carvajal AJ, Kwong JS (2016). Pharmacological interventions for treating heart failure in patients with Chagas cardiomyopathy. Cochrane Database Syst Rev.

[98] Bocchi EA Guimarães GV, Argentina, Chile, and Brazil SHIFT Investigators (2018). Safety profile and efficacy of ivabradine in heart failure due to Chagas heart disease: a post hoc analysis of the SHIFT trial. ESC Heart Fail.

[99] Ramires FJA, Martinez F, Gómez EA, Demacq C, Gimpelewicz CR, Rouleau JL (2018). Post hoc analyses of SHIFT and PARADIGM-HF highlight the importance of chronic Chagas' cardiomyopathy Comment on "Safety profile and efficacy of ivabradine in heart failure due to Chagas heart disease: a post hoc analysis of the SHIFT trial" by Bocchi et al. ESC Heart Fail.

[100] Piepoli MF, Conraads V, Corrà U, Dickstein K, Francis DP, Jaarsma T (2011). Exercise training in heart failure from theory to practice. A consensus document of the Heart Failure Association and the European Association for Cardiovascular Prevention and Rehabilitation. Eur J Heart Fail.

[101] Mendes FSNS, Mediano MFF, Souza FCC, Silva PS, Carneiro FM, Holanda MT (2020). Effect of physical exercise training in patients with Chagas heart Disease (from the PEACH STUDY). Am J Cardiol.

[102] Carod-Artal FJ (2007). Stroke a neglected complication of American trypanosomiasis (Chagas' disease). Trans R Soc Trop Med Hyg.

[103] Oliveira JSM, Araujo RRCD, Navarro MA, Muccillo G (1983). Cardiac thrombosis and thromboembolism in chronic Chagas' heart disease. Am J Cardiol.

[104] Cardoso RN, Macedo FYB, Garcia MN, Garcia DC, Benjo AM, Aguilar D (2014). Chagas cardiomyopathy is associated with higher incidence of stroke a meta-analysis of observational studies. J Card Fail.

[105] Sousa AS, Xavier SS, Freitas G, Hasslocher-Moreno AM (2008). Prevention strategies of cardioembolic ischemic stroke in Chagas' disease. Arq Bras Cardiol.

[106] Mendes FSNS, Mediano MFF, Silva RS, Xavier SS, do Brasil PEAA, Saraiva RM (2020). Discussing the score of cardioembolic ischemic stroke in Chagas disease. Trop Med Infect Dis.

[107] Monteiro JMC, San-Martin DL, Silva BCG, de Jesus PAP, Oliveira J (2018). Anticoagulation in patients with cardiac manifestations of Chagas disease and cardioembolic ischemic stroke. Arquivos de Neuro-Psiquiatria.

[108] Dias JC, Ramos AN, Gontijo ED, Luquetti A, Shikanai-Yasuda MA, Coura JR (2016). 2nd Brazilian consensus on Chagas disease, 2015. Rev Soc Bras Med Trop.

[109] Martinelli M, Rassi A, Marin-Neto JA, de Paola AA, Berwanger O, Scanavacca MI (2013). Chronic use of Amiodarone against Implantable cardioverter-defibrillator therapy for primary prevention of death in patients with Chagas cardiomyopathy study: rationale and design of a randomized clinical trial. Am Heart J.

[110] Gali WL, Sarabanda AV, Baggio JM, Ferreira LG, Gomes GG, Marin-Neto JA (2014). Implantable cardioverter-defibrillators for treatment of sustained ventricular arrhythmias in patients with Chagas' heart disease comparison with a control group treated with amiodarone alone. Europace.

[111] Cardinalli-Neto A, Bestetti RB, Cordeiro JA, Rodrigues VC (2007). Predictors of all-cause mortality for patients with chronic Chagas' heart disease receiving implantable cardioverter defibrillator therapy. J Cardiovasc Electrophysiol.

[112] Carmo AAL, de Sousa MR, Agudelo JF, Boersma E, Rocha MOC, Ribeiro ALP (2018). Implantable cardioverter-defibrillator in Chagas heart disease a systematic review and meta-analysis of observational studies. Int J Cardiol.

[113] Scanavacca MI, Sosa EA, Lee JH, Bellotti G, Pileggi F (1990). Empiric therapy with amiodarone in patients with chronic Chagas cardiomyopathy and sustained ventricular tachycardia. Arq Bras Cardiol.

[114] Sosa E, Scanavacca M, D'Avila A.Bellotti G.Pilleggi F (1999). Radiofrequency catheter ablation of ventricular tachycardia guided by nonsurgical epicardial mapping in chronic Chagasic heart disease. Pacing Clin Electrophysiol.

[115] Henz BD, Nascimento TA, Dietrich CO, Dalegrave C, Hernandes V, Mesas CE (2009). Simultaneous epicardial and endocardial substrate mapping and radiofrequency catheter ablation as first-line treatment for ventricular tachycardia and frequent ICD shocks in chronic chagasic cardiomyopathy. J Interv Card Electrophysiol.

[116] Pisani CF, Romero J, Lara S, Hardy C, Chokr M, Sacilotto L (2020). Efficacy and safety of combined endocardial/epicardial catheter ablation for ventricular tachycardia in Chagas disease a randomized controlled study. Heart Rhythm.

[117] Bocchi EA, Bellotti G, Mocelin AO, Uip D, Bacal F, Higuchi ML (1996). Heart transplantation for chronic Chagas heartdisease. Ann Thorac Surg.

[118] Costa PA, Segatto M, Durso DF, Moreira WJC, Junqueira LL, Castilho FM (2017). Early polymerase chain reaction detection of Chagas disease reactivation in heart transplant patients. J Heart Lung Transplant.

[119] Bocchi EA, Fiorelli A (2001). The paradox of survival results after heart transplantation for cardiomyopathy caused by Trypanosoma cruzi First Guidelines Group for Heart Transplantation of the Brazilian Society of Cardiology. Ann Thorac Surg.

[120] Andrade JP, Marin JA, Paola AA, Vilas-Boas F, Oliveira GM, Bacal F (2011). Latin American guidelines for the diagnosis and treatment of Chagas' heart disease executive summary. Arq Bras Cardiol.

[121] Bocchi EA, Fiorelli A (2001). First guideline group for heart transplantation of the Brazilian Society of Cardiology the Brazilian experience with heart transplantation: a multicenter report. J Heart Lung Transplant.

[122] Lee BY, Bacon KM, Bottazzi ME, Hotez PJ (2013). Global economic burden of Chagas disease a computationals imulation model. Lancet Infect Dis.

[123] Kransdorf EP, Zakowski PC, Kobashigawa JA (2014). Chagas disease in solid organ and heart transplantation. Curr Opin Infect Dis.

[124] Mehra MR, Canter CE, Hannan MM, Semigran MJ, Uber PA, Baran DA (2016). The 2016 International Society for Heart Lung Transplantation listing criteria for heart transplantation a 10-year update. J Heart Lung Transpl.

[125] Parra AV, Rodrigues V, Cancella S, Cordeiro JA, Bestetti RB (2008). Impact of socioeconomic status on outcome of a Brazilian heart transplant recipients cohort. Int J Cardiol.

[126] Costanzo MR, Dipchand A, Starling R, Anderson A, Chan M, Desai S (2010). The International Society of Heart and Lung Transplantation Guidelines for the care of heart transplant recipients. J Heart Lung Transplant.

[127] Bacal F, Silva CP, Bocchi EA, Pires PV, Moreira LF, Issa VS (2005). Mychophenolate mofetil increased Chagas disease reactivation in heart transplanted patients comparison between two different protocols. Am J Transplant.

[128] Fiorelli AI, Santos RH, Oliveira JL, Lourenço-Filho DD, Dias RR, Oliveira AS (2011). Heart transplantation in 107 cases of Chagas' disease. Transplant Proc.

[129] Godoy HL, Guerra CM, Viegas RF, Dinis RZ, Branco JN, Neto VA (2010). Infections in heart transplant recipients in Brazil the challenge of Chagas' disease. J Heart Lung Transplant.

[130] Camargos S, Moreira MDV, Portela DMMC, Imperes Lira JP, Santos FVM, Menezes GMM (2017). CNS chagoma reactivation in an immunosuppressed patient. Neurology.

[131] de Souza MM, Franco M, Almeida DR, Diniz RV, Mortara RA, da Silva S (2001). Comparative histopathology of endomyocardial biopsies in chagasic and non chagasic heart transplant recipients. J Heart Lung Transplant.

[132] Shikanai-Yasuda MA, Carvalho NB (2012). Oral transmission of Chagas disease. Clin Infect Dis.

[133] MS, SVS (2015). Boletim Epidemiológico.

[134] Steverding D (2014). The history of Chagas disease. Parasit Vectors.

[135] Pinto AY, Ferreira AG, Valente V, Harada GS, Valente AS (2009). Urban outbreak of acute Chagas disease in Amazon region of Brazil four-year follow-up after treatment with benznidazole. Rev Panam Salud Publica.

[136] Nóbrega AA, Garcia MH, Tatto E, Obara MT, Costa E, Sobel J (2009). Oral transmission of Chagas disease by consumption of açaí palm fruit, Brazil. Emerg Infect Dis.

[137] Barreto-de-Albuquerque J, Silva-dos-Santos D, Pérez AR, Berbert LR, de Santana-van-Vliet E, Farias-de-Oliveira DA (2015). Trypanosoma cruzi infection through the oral route promotes a severe infection in mice new disease form from an old infection?. PLoS Negl Trop Dis.

[138] de Góes Costa E.Dos Santos SO.Sojo-Milano M.Amador EC.Tatto E.Souza DS (2017). Acute Chagas disease in the Brazilian Amazon epidemiological and clinical features. Int J Cardiol.

[139] Vazquez BP, Vazquez TP, Miguel CB, Rodrigues WF, Mendes MT, de Oliveira CJ (2015). Inflammatory responses and intestinal injury development during acute Trypanosoma cruzi infection are associated with the parasite load. Parasit Vectors.

[140] Souza DSM, Araujo MT, Garcez PS, Furtado JC, Fiqueiredo MT, Povoa RM (2016). Aspectos anatomopatológicos da miocardite chagásica agudo por transmissão oral. Arq Bras Cardiol.

[141] Rassi A, Rassi A, Marin-Neto JA (2010). Chagas disease. Lancet.

[142] Pinto AY, Valente SA, Valente VC, Ferreira AG, Coura JR (2008). Fase aguda da doença de Chagas na Amazônia brasileira estudo de 233 casos do Pará, Amapá e Maranhão observados entre 1988 e 2005. Rev Soc Bras Med Trop.

[143] Souza DS, Almeida AJ, Costa FA, Costa EG, Figueiredo MT, Póvoa RM (2013). O eletrocardiograma na fase aguda da doença de Chagas por transmissão oral. Rev Bras Cardiol.

[144] Souza DSM, Povoa RMS (2016). Aspectos epidemiológicos e clínicos da doença de Chagas aguda no Brasil e na América Latina. Rev Soc Cardiol Estado de São Paulo.

[145] Souza DSM, Oliveira CB, Maciel BG, Figueiredo MT, Bianco HT, Fonseca FAH (2020). Absence of nocturnal fall in blood pressure detected by ambulatory blood pressure monitoring in acute Chagas disease patients with oral infection. Arq Bras Cardiol.

[146] Roggero E, Pérez AR, Pollachini N, Villar SR, Wildman J, Besedovsky H (2016). The sympathetic nervous system affects the susceptibility and course of Trypanosoma cruzi infection. Brain Behav Immun.

[147] Póvoa MM, de Souza AA, Naiff RD, Arias JR, Naiff MF, Biancardi CB (1984). Chagas' disease in the Amazon basin IV Host records of Trypanosoma cruzi zymodemes in the states of Amazonas and Rondonia, Brazil. Ann Trop Med Parasitol.

[148] CONITEC (2018). Protocolo clínico e diretrizes terapêuticas doença de Chagas. Relatório de recomendação.

[149] Santos VRC, Antunes D, Souza DSM, Moreira OC, Lima ICA, Farias-de-Oliveira DA (2020). Human acute Chagas disease changes in factor VII, activated protein C and hepatic enzymes from patients of oral outbreaks in Pará State (Brazilian Amazon). Mem Inst Oswaldo Cruz.

[150] Compagnucci AB, Ddvila A, Beloscar J, Pezzotto SM, Davila H (2016). Dietary intake and nutritional status of patients with Chagas disease. Arch Latinoam Nutr.

[151] Malachias MVB, Souza WKSB, Plavnik FL, Rodrigues CIS, Brandão AA, Neves MFT (2016). 7th Brazilian Guideline of Arterial Hypertension. Arq Bras Card.

[152] Tarasoutchi F, Montera MW, Ramos AIO, Sampaio RO, Rosa VEE, Accorsi TAD (2017). Atualização das diretrizes brasileiras de valvopatias. Abordagem das lesões anatomicamente importantes. Arq Bras Cardiol.

[153] Faludi AA, Izar MCO, Saraiva JFK, Chacra APM, Bianco HT, Afiune A (2017). Dislipidemias e prevenção da aterosclerose-2017. Arq Bras Cardiol.

[154] Medeiros CA, Martins SM, da Silva TFL, da Silva PM, Carrazzone CFV, Barros MNDS (2019). Perfil dos portadores de doença de Chagas no século XXI: comorbidades. In 55º Congresso da SBMT | CHAGASLEISH. Anais do 26º Congresso Brasileiro de Parasitologia. UFMG.

[155] Vizzoni AG, Varela MC, Sangenis LHC, Hasslocher-Moreno AM, do Brasil PEAA, Saraiva RM (2018). Ageing with Chagas disease an overview of an urban Brazilian cohort in Rio de Janeiro. Parasit Vectors.

[156] Lage SG, Mansur AP, Ramires JA, da Luz P, Bellotti G, Pileggi F (1986). Acute myocardial infarction in chronic Chagas' cardiomyopathy Report of two cases with no obstructive coronary artery lesions. Rev Inst Med Trop São Paulo.

[157] Torres FW, Acquatella H, Condado JA, Dinsmore R, Palacios IF (1995). Coronary vascular reactivity is abnormal in patients with Chagas' heart disease. Am Heart J.

[158] Marin-Neto JA, Marzullo P, Marcassa C, Gallo L, Maciel BC, Bellina CR (1992). Myocardial perfusion abnormalities in chronic Chagas'disease as detected by thallium-201 scintigraphy. Am J Cardiol.

[159] Neder JA, Rocha A, Alencar MCN, Arbex F, Berton DC, Oliveira MF (2018). Current challenges in managing comorbid heart failure and COPD. Expert Rev Cardiovasc Ther.

[160] Liao KM, Lin TY, Huang YB, Kuo CC, Chen CY (2017). The evaluationof ß-adrenoceptor blocking agents in patientswith COPD and congestive heart failure a nation wid estudy. Int J Chron Obstruct Pulmon Dis.

[161] Dauriz M, Targher G, Laroche C, Temporelli PL, Ferrari R, Anker S (2017). Associationbetween diabetes and 1-year adverse clinical outcomes in a multinational cohort of ambulatory patient with chronic heart failure: result from the ESC-HFA Heart failure Long-Term Registry. Diabetes Care.

[162] Dunlay SM, Givertz MM, Aguilar D, Allen LA, Chan M, Desai AS (2019). Type 2 diabetes mellitus and heart failure a scientific statement from the American Heart Association and the Heart Failure Society of America: this statement does not represent an update of the 2017 ACC/AHA/HFSA heart failure guideline update. Circulation.

[163] McMurray JJV, Solomon SD, Inzucchi SE, Køber L, Kosiborod MN, Martinez FA (2019). Dapagliflozin in patients with heart failure and reduced ejection fraction. N Engl J Med.

